# Using contraceptives to delay first birth: a qualitative study of individual, community and health provider perceptions in southern Tanzania

**DOI:** 10.1186/s12889-017-4759-9

**Published:** 2017-10-03

**Authors:** Yovitha Sedekia, Caroline Jones, Rose Nathan, Joanna Schellenberg, Tanya Marchant

**Affiliations:** 1 Ifakara Health Institute, P.O BOX 78373, Dar-es-Salaam, Tanzania; 2 Department of Disease Control, London School of Hygiene and Tropical Medicine, Keppel Street, London, WC1E 7HT UK; 3 KEMRI-Wellcome Trust Research Programme, Kilifi, Kenya

**Keywords:** Acceptability, Modern contraceptives, Delayers of first birth, Family planning use, Maternal and child health, Tanzania

## Abstract

**Background:**

Young adolescents and unmarried women in low and middle income countries face challenges in accessing family planning services. One factor likely to limit contraceptive use is the attitude and opinion of local stakeholders such as community leaders and health workers. Much of the existing evidence on this topic focuses on women who have already started childbearing. Using primary qualitative data, we explored individual, community and health provider’s perceptions about using modern contraceptives to delay the first birth in a high fertility setting.

**Methods:**

A descriptive qualitative study was conducted in Tandahimba district in southern Tanzania between December 2014 and March 2015. We conducted 8 focus group discussions with men and women and 25 in-depth interviews (18 with women, 4 with family planning service providers and 3 with district-level staff). Participants were purposively sampled. Data transcripts were managed and coded using Nvivo 11 software and we employed a thematic framework analysis.

**Results:**

Three main themes emerged about using modern contraceptives to delay first birth: (1) the social and biological status of the woman (2) the type of contraceptive and (3) non-alignment among national policies for adolescents. Use of modern contraceptives to delay first birth was widely acceptable for women who were students, young, unmarried and women in unstable marriage. But long-acting reversible methods such as implants and intrauterine devices were perceived as inappropriate methods for delaying first birth, partly because of fears around delayed return to fecundity, discontinuation once woman’s marital status changes and permanently limiting future fertility. The support for use of modern contraceptives to delay a first pregnancy was not unanimous. A small number of participants from both rural and urban areas did not approve the use of contraceptive methods before the birth of a first baby at all, not even for students. There was lack of clarity and consistency on the definition of ‘young’ and that had direct implications for access, autonomy in decision-making, confidentiality and consent for young people.

**Conclusions:**

Women who wish to delay their first birth face challenges related to restrictions by age and method imposed by stakeholders in accessing and provision of modern contraceptives. There is a need for a clearly communicated policy on minimum age and appropriate method choice for delayers of first birth.

## Background

Globally in 2015, 57% of married or in-union women of reproductive age were using modern contraception, but the use was much lower in the least developed countries (34%) and was particularly low in Sub-Saharan Africa (24%) [1]. Modern contraceptive use among adolescents is effective at helping to prevent teenage pregnancy which in turn leads to averting abortions, miscarriage and maternal and newborn deaths [2, 3]. The use of modern contraceptives also helps the adolescents to complete and attain longer education, a later and healthier start to child birth and more opportunity to engage in income generating activities [2–6]. In low and middle income countries some adolescent girls are sexually active before the age of 15 years [7–9], age at first marriage is increasing [10], contraceptive use among married or in union women is increasing [1] and fertility is declining. At the same time, education and the right to determine timing of a pregnancy is becoming more prevalent among women [11, 12]. While education had been reported to have an effect on increasing age at first marriage [13], the combination of the initiation of sexual activities even before the age of 15 years and increasing age of first marriage lead to the possibility that more young women are at risk of early or unplanned first pregnancy if they do not have adequate access to contraception. It is important to understand the factors that enhance or constrain contraceptive use among this group. A recent analysis of DHS data from 52 developing countries found that a large proportion of women (married and unmarried) cite fear of contraceptive side effects and infrequent sex as reasons for not using contraception, and among unmarried women, simply being unmarried was one of the reasons for not using family planning [14]. In addition, the attitudes and opinions of local stakeholders such as community leaders and health workers have been found to limit contraceptive use among young and unmarried women in developing countries [8, 15–21], as well as women being pressured to prove their fertility as soon as they get married [22, 23].

Family Planning 2020 is an initiative to expand contraceptive use to 120 million additional women and girls by 2020 [24, 25]. Tanzania is one of the Family Planning 2020 focus countries and has the policy, strategy and guidelines in place to respond to this initiative. All men and women in the country, including young people (10–24 years of age) regardless of parity, marital status, creed, race, or sexual preference are eligible to access family planning information, education and services. There is no parental, guardian or spousal consent required for adolescents (10–18 years), although such consent is required for people with severe mental disabilities and clients seeking permanent methods [26, 27]. As the legal minimum marriage age for women in Tanzania is 15 years with parental consent [28], this policy allows for the provision of family planning services to young unmarried women. Modern contraceptive methods approved for use in Tanzania include: implants, male and female sterilization, oral contraceptive pills, progestogen-only injectables, intrauterine device - copper containing only (IUCD), male and female condoms and emergency oral contraceptive pills [26]. In addition, the government is committed to making family planning services accessible, safe, acceptable, affordable, and encourages integration or linkage with other reproductive and child health services [26, 27]. In 2015, 32% of currently married or in union women aged 15–49 years in Tanzania were using modern contraceptive methods lower than estimates for sexually active unmarried women (46%) [29], and for Eastern Africa (40%) [1] for the same period. The use was much lower among adolescent girls aged 15–19 years (9%) [29].

While the strategies, guidelines and policy frameworks are in place, little is known about how they are being implemented, including the opportunities and challenges that exist in accessing modern contraceptives among women wanting to delay their first pregnancy. Using primary qualitative data from the high fertility setting of Tanzania, we explored individual, community and health provider’s perceptions about the acceptability of modern contraceptives to delay first birth.

## Methods

### Study setting

The study was conducted in Tandahimba district in southern Tanzania. Tandahimba district covers an estimated population of 227,500 people served by 34 health facilities (33 primary care facilities and one hospital) [30, 31]. In 2015, 87% of health facilities were offering any modern method of family planning, 58% had samples of family planning methods in stock, and only 18% had at least one staff trained in family planning services [32]. Characterised as a predominantly rural area with limited infrastructure, over 90% of the population depends on agricultural activities especially cashew nuts for their subsistence [33]. The area has a total fertility rate of 4 and a median age at first birth of 19 years [29]. Estimates of modern contraceptive use are high compared to Tanzanian mainland at 50% among currently married women (32% mainland) and 70% among sexually active unmarried women aged 15 to 49 years (46% mainland) [29], but the neonatal and maternal mortality rates are also high at 47 newborn deaths per 1000 live births [29] and 712 per 100,000 live births [34] respectively. A study in southern Tanzania reported that four percent of all sexually active women aged 13–49 years wanted to delay their first birth and this group of women overlap substantially, but not exclusively, with the adolescent age group (10–19 years) [35]. Forty one percent of these women who wanted to delay their first pregnancy had unmet need for modern contraceptives [35]. The implementation of family planning services in the study area is guided by the national policy, strategies and guidelines [26, 27].

### Study design and data collection

This was a descriptive, qualitative study. Between December 2014 and March 2015, we conducted eight focus group discussions (FGDs) with men and women, 25 in-depth interviews (IDIs): 18 with women, four with family planning service providers and three with district-level staff.

#### Focus group discussions

Eight FGDs, four in urban settings and four in rural settings with 6–12 participants per group were conducted. In each setting one FGD was held with each of the following: men above 20 years; men below 20 years; women above 20 years; and women below 20 years. Participants were purposively sampled from the community to reflect the range of people living in the community, and a total of 71 people took part in the FGDs (Table 1). A topic guide covering issues such as: use of contraceptives in their communities; community acceptability of modern contraceptives for women who would like to delay their first birth; and type of contraceptive methods appropriate for the delayers, was used to guide the discussions. Each discussion lasted between 60 and 90 min. Women’s FGDs were facilitated by the female principal investigator (YS) and the FGDs with men were facilitated by an experienced male research assistant who also received a five days training that included pilot testing of the topic guides specifically for this study. Four FGDs were conducted in the village executive offices in their respective villages and four were conducted in a classroom at one of the primary schools in their area.

**Table 1:** Characteristics of participants in community focus group discussion and in-depth interview

Background characteristics	Type of respondent			
	FGD participants (men) *N* = 38	FGD participants (women) *N* = 33	IDIs with women in the community who were current users of modern contraceptives (*N* = 9)	IDIs with women in the community who were current non-users of modern contraceptives (*N* = 9)
Age				
13–19	15	16	5	5
20–39	15	16	2	2
40 and above	8	1	2	2
Marital status				
Single	16	16	5	5
Currently Married/cohabiting	20	15	4	4
Divorced	2	2	0	0
Marriage type^¥^				
Monogamous	17	≠	1	1
Polygamous	3	≠	3	3
Area of residence				
Urban	21	15	5	5
Rural	17	18	4	4
Number of living children				
None	22	15	5	5
1	7	6	0	3
2–4	7	10	4	1
> 4	2	2	0	0
Number of marriages a divorced or currently married woman has had				
1	–	8	3	2
2	–	7	1	1
3	–	2	0	1
Number of wives a divorced or currently married men has had				
1	18	–	–	–
2	3	–	–	–
3	1	–	–	–

¥ for currently married/cohabiting; −Not applicable; ≠ Information was not requested from the participants

#### In-depth interviews

We conducted 18 semi-structured in-depth interviews with women who did not take part in FGDs (Table 1). The women were purposively sampled from the community based on their age (above or below 20 years), use of modern contraceptives (current use or non-use), and place of residence (urban or rural) to reflect the range of women living in the community. Women were asked about eligibility to use contraceptives; if they approve use of modern contraception to delay first birth; appropriate types of contraceptive methods for delayers of first birth; and if their husbands or partners approve use of contraceptives to delay first birth. The interviews were conducted in a place preferred by the respondent, most often her home.

We conducted four semi-structured in-depth interviews with family planning service providers (Table 2). The providers were purposively selected from different types and levels of health facilities (one from each of hospital, public health centre, mission health centre, and a rural dispensary). Where there was more than one family planning provider employed we purposively selected the staff member allocated to provide services on the day of interview (one from nine family planning providers employed at the hospital, one from six family planning providers employed at the public health centre and one from two family planning providers employed at the mission health centre. The rural dispensary employed only one staff member for all services. The service providers were also asked about: eligibility to receive modern contraceptive methods and the methods that they recommend for women who would like to delay their first birth. Interviews with the service providers were conducted in their offices early in the morning before their clients started coming for services or late afternoon when the staff had finished attending all clients.

**Table 2:** Characteristics of family planning providers and district stakeholders participating in in-depth interviews

Background characteristics	Type of respondent	
	IDIs with health facility family planning service providers (*N* = 4)	IDIs with district-level staff (*N* = 3)
Cadre		
District medical officer	–	1
DRCHco	–	1
DHSWO	–	1
Enrolled nurse	2	–
Registered nurse	1	–
Medical attendant	1	–
Number of years working at the facility		
< 1 year	1	1
1–10 years	2	–
> 10 years	1	2
Years providing/overseeing FP services at the facility		
< 1 year	1	1
1–10 years	2	–
> 10 years	1	2

- not applicable; *DRCHco* District Reproductive and Child Health co-ordinator, *DHSWO* District Hospital Social Welfare Officer

We conducted semi-structured interviews with: 1) one District Medical Officer (DMO); 2) one District Reproductive and Child Health coordinator (DRHco) who oversees reproductive and child health services in the district; and 3) one District Hospital Social Welfare Officer (DHSWO) (Table 2). In-depth interviews with DMO and DRCHco asked about provision of family planning services and policy and guidelines that govern the service provision in their district. The DHSWO was interviewed to understand his involvement in family planning services in the district.

All IDIs were facilitated by the female principal investigator and lasted for up to one hour. Village or street leaders assisted in the sampling of FGD and IDIs participants in their communities.

### Data management and analysis

The FGDs and interviews were all conducted in Swahili language and recorded and transcribed verbatim. A few transcribed interviews were translated into English and shared with a non- Swahili speaker involved in the study (TM) for comments during the initial stage of data collection. Results were translated from Swahili to English by the principal investigator during the final interpretation of the data presented in this study. Data analysis took place alongside data collection. Data transcripts were managed and coded using NVivo 11 software. We employed a thematic framework analysis method [36] in which themes were derived from the research questions as well as emerging from the discussion and interview data [37]. The analysis included five steps. The first step involved familiarization with the data during which the principal investigator listened to the audio recordings and read through the field notes and transcripts. The second step involved coding a few transcripts and identifying initial themes which were shared with TM, discussed, and the work refined until no new themes were generated. All data were coded by the principal investigator. The third step involved organising codes reflecting prominent themes within the data set. The fourth step involved creating a framework matrix for each theme and charting data for each code, IDIs and FGDs within that theme (comparison of both within and between interviews), and fifth step involved data mapping and interpretation through reviewing the matrices and looking at relationships between the codes and the interviews (IDIs & FGDs) [38, 39].

## Results

The characteristics of the study participants are shown in Tables 1 and 2. A total of 71 participants took part in FGDs (38 men and 33 women). About half of men and women participants were currently married. More than half of women participants who were currently married had been married more than once; − i.e. they were in their second or third marriages. More than half of male had never had a child and about half of women had at least one child. Half of IDIs participants (current users and non-users of contraception) had never had a child and majority of currently married women were in polygamous marriage. All current users of modern contraceptives who had started childbearing had more than one child whereas, more than half of non-users of contraception had one child (Table 1).

Of the four family planning service providers interviewed, two were enrolled nurses, one a registered nurse and one a medical attendant. The majority of the service providers and district-level staff had worked and provided family planning services in their current facilities for more than one year (Table 2).

Across all group types, three main emerging themes from the analysis about using modern contraceptives to delay first birth were: (1) the social and biological status of the woman (2) the type of contraceptives and (3) non-alignment among national policies for adolescents.

### The social and biological status of the woman

There was strong consensus among participants across all group types that some groups of sexually active women should use modern contraceptives to delay their first birth. These were: students, young women, unmarried women, and women in unstable marriage.

#### Being a student

It was commonly agreed across all groups that using modern contraceptives to delay first birth was acceptable and widely used among students in order to avoid pregnancies while they were still studying.

*“Yeah, they approve, especially we young generation because many of we youth who are in secondary schools use modern contraceptives to avoid unwanted pregnancies…..” (FGD, 18 year-old man, rural).*

A variety of justifications were given. Firstly, across all groups the importance of secondary education for young people was raised: *“We need a large percentage of our children to be educated ……… The education will help her or him to get good earning” IDI01, 47 years-old woman, currently non-user of modern contraceptives, urban)* and pregnancy was widely perceived to impact on the ability of a young woman to complete her secondary education as schools reportedly take punitive action against pregnant students.

*“……once you become pregnant they tell you that’s the end of your studies…………. (IDI05, 16 years-old woman, currently non-user of modern contraceptives, urban).*

Furthermore, there were concerns that, in addition to the girl being expelled from school, schools might invoke legal action against the girl’s parents or partner.

*“…..first of all, some [parents] are taken to the court if she [student] becomes pregnant while she is still studying….the school take parents to the court…….”* (IDI03, FP service provider, dispensary, rural).

The issue of a student’s mother being blamed for her daughter’s pregnancy was also raised as a concern by some family planning services providers and several FGD participants of both sexes aged 20 years and above from rural area.

*“…..Eeh, it happened in our family as well, my sister was in secondary school. She became pregnant while at school, my father accused my mother to be the source of my sister’s pregnancy by saying that she [=my sister] is so close to her mother, she turns to her for everything (FGD, 30 years-old man, rural).*

These data suggest that families are concerned about the economic, legal and social consequences of a studying daughter becoming pregnant and it was suggested by several participants that families played a role in facilitating access to modern contraceptives for female students in their households.

*“….in some households they take their children to the dispensary for implants or pills and they complete their studies without any problem. But in some households [where they do not] the father becomes furious when it happens [=his daughter getting unwanted pregnancy]……” (FGD, 26 years-old man, rural).*

*“What I have learnt from students using contraceptives is that, first of all, parents themselves are the one who bring the students….[they say] aaah, nowadays these children behave badly, they might get pregnant before completing their studies” (IDI03, FP service provider, dispensary).*

*“...I have a [girl] child who has completed standard seven [=primary school education]…...I will take her [to the facility for contraception] when she is nearly going to join [secondary] school……” (FGD, 38 years-old woman, urban).*

#### Being young

Participants reported that it was also acceptable for young married and unmarried women to use modern contraceptives to delay their first birth. Participants however, held mixed views regarding the age that a woman was considered “young”. There was no commonly agreed age for being considered “young” or at first use of contraception across all groups but they agreed that girl’s age at first sex had decreased and many girls started sexual activities while they were still studying, and thus justifying the use of modern contraception.

*“From twelve years because nowadays many children start engaging into sexual activities while they are still at school…. (FGD, 37 years-old woman, rural).*

***“*** *…….sixteen years-old girl should use contraceptives because at that age many children are at school and start engaging [into sex] while they have not completed their studies..... (FGD, 19 years-old woman, rural).*

*“…nowadays even 13 years old girls come for contraceptive method, though not many [the 13 years girls] in this village [who come], but 13, 14, 16 years [girls], students from standard five and above come to access contraception (IDI03, FP service provider, dispensary).*

And some start sexual activities even before getting their first menstrual period or they experience teenage marriage:

***“*** *Nowadays I approve [use of contraceptive to delay first birth] because many children start engaging into love affairs at a very young age unlike during our days..... Nowadays children start sexual intercourse even before getting their first period….” (IDI01 47 years-old woman, currently non-user of modern contraceptives, rural).*

*“….sometimes you find a 14 years girl get married. That is a very young age. It is better for her to delay her first birth until she at least turns 16 or 18 years old. [At that age] she is at least matured enough “kuhimili vishindo” [=to cope with challenging situations] (FGD, 19 years-old man, urban).*

Several, but not all participants expressed the view that early pregnancies were bad for girl’s health and use of modern contraceptives by young people was justifiable.

*“From 12 years, a girl should delay her first birth until she at least turns 17 years or 18 years old to avoid teenage pregnancy…..It [=teenage pregnancy] affects her during delivery as she may not get complete cervical dilatation. She is not matured enough. She has to use pills or injectables or implants until when she reaches a reproductive age.” (FGD, 26 years old woman, urban).*

*“…..So, since they [=girls] are allowed to marry from the age of 15 years, you cannot just let them be pregnant at that age [=by denying them contraception]. Their reproductive organs are not matured enough for pregnancy. She has to delay a bit until she at least turns 18 years” (IDI05, DRCHco).*

Conversely, some participants from both rural and urban areas expressed fear of infertility and did not approve use of modern contraceptives for women below the age of 18 years.

*“……..when you use before giving birth [to a child] the uterus gets affected, it rots, and fallopian tubes lose the capacity to hold the ovary. So, you become the source of your infertility (* ***I*** *: from what age?) 15 to 17 years” (FGD, 13 years-old girl, rural).*

***“*** *One day I saw a certain woman at the hospital. The doctor [=service provider] asked her, “you woman, are you mentally fit? You brought such a young child for injectables or implants? Do you know that you are causing her to be infertile?”......I do not know if they were unfair to her or not, but I saw her” (FGD, 36 years-old woman, urban).*

#### Being unmarried

The majority of participants across all FGDs (men and women) and IDIs with women reported use of contraceptives to delay first birth to be acceptable for unmarried women. Two key reasons given were both associated with the social and economic consequences of being an unmarried mother. First, a pregnancy outside of marriage brought shame to the woman, and this social stigma may transfer to the family more broadly.

***“….*** *if she [unmarried woman] does not use [contraceptive methods] she will become pregnant and get illegitimate child. It is a shame. To avoid an embarrassment she has to use contraceptive methods” (FGD, 45 years-old man, urban).*

Second, it was said to be easier for a man to abandon a pregnant woman if they were not married; leaving the woman vulnerable both economically and socially, or alternatively being forced by her parents to go and live with the man’s family even before marriage.

***“……*** *...aah, other men abandon you. So, once he abandons you, you remain with your pregnancy on your own. Parents also do not like it. (IDI01, 16 years-old woman, contraceptive user of modern contraceptive, urban).*

*“He impregnated me, and then my parents asked who was responsible with the pregnancy, I told them. I and my parents went to his family to meet with his parents. They asked him and he agreed that he had sexual affairs with me and the pregnancy was his. Then I was left there [=to stay into his family’s home], he and his parents were the ones taking care of me…….Eeh, it was before we got married, I only had the pregnancy ……………” (IDI02, 20 years-old woman, currently non-user of modern contraceptives, rural).*

Despite the social stigma and economic consequences of being an unmarried mother, several respondents across all groups were of the view that pregnancies outside marriage were relatively common in the study area.

*“Mmh! It is a common thing [=for unmarried women to become pregnant] in this area because majority who have given birth are not married. There are so many children born out-of-wedlock……” (IDI02, 20 years-old woman, currently non-user of modern contraceptives, rural).*

#### Being married in an unstable marriage

Participants across all FGDs and IDIs had mixed views about delaying first birth for married women. A few participants across all groups reported that it was acceptable for married women to delay their first birth only if they were not quite sure about their husband’s behaviours and needed to first understand them better before they started child bearing.

***“*** *You find a woman who marries and has no child within a year since marriage. When you ask her what happened she replies, “Such a man, can you just get him a child so soon? I have to understand his behaviours”. Some men are “bondia”*” *(=they beat their wives) (FGD, 36 years-old man, urban).*

This was corroborated by health facility providers who said they receive both married and unmarried women who come for modern contraceptives in order to delay their first birth.

*“……for the few [married women] that I have received told me that “I still don’t know his behaviour. So, I cannot just get married and become pregnant. I would like to use contraceptives for two years then after we have known each others’ behaviour better, I can decide to have a child…..” (IDI01, FP service provider, district hospital).*

It was also acceptable if a woman was married to an uncommitted and financially unstable man.

***“*** *Some [women] do not stop [using modern contraceptives]. She first observe economic situation of the household she is married in......So my opinion is the same- to first delay for at least six months while observing the situation” (FGD, 19 years-old woman, urban).*

However, the majority of the participants across all groups held the view that married men or even some married women do not accept using contraceptives for delaying first birth because they want to start having children.

*“*. …*.once I have married her and she tells me that she wants to delay first pregnancy, I will not agree and I cannot live with such a woman …..” (FGD, 45 years-old man, urban).*

The importance of having children to raise an individual’s status in the community was very prevalent.

*“Children are very important in the community in various situations. If you do not have a child, you are not a rich person. Even if you are a millionaire, you are nothing because a child can help you in many challenges and without a child you can get a lot of problems in this world” (FGD, 45 years-old man, urban).*

In addition a view was expressed that the rise of more nuclear style families made social and economic reliance on children rather than family clans even more important.

***“*** *…..the clan era has ended. Therefore you and your child have to rely on each other and not the society….” (IDI01, 24 years-old woman, current user of modern contraceptive, rural).*

Although families and children were viewed as important and the Islam which is the dominant religious belief in the study area does not allow use of modern contraception, participants across all FGDs (with men and women) and IDIs with women commonly acknowledged that nevertheless people have decided to use them.

*“According to religious beliefs it is not allowed to use modern contraceptives, but the community has decided, that is why we use contraceptives” (FGD, 39 years-old woman, rural).*

#### Divergent views

The support for use of modern contraceptives to delay a first pregnancy of a student, a young person, unmarried, or married in unstable marriage was not unanimous. A small number of participants from both rural and urban areas held different views, with some not approving the use of contraceptive methods before the birth of a first baby at all, not even for students.

*“Family planning is not for delaying first birth. The aim of family planning [methods] is to space between the first child and subsequent child”* (*FGD, 18 years old man, rural).*

The main reason for disapproval was based on the “defined fertility concept” that a woman has a limited fertility and if contraceptive methods were used, a woman might end up with infertility.

*It might happen that God has destined the person to have a single child and at a certain specific time. It might happen that the person has one or two eggs [= this is about fertility/fecundity], once she uses contraceptive methods and destroy the eggs will she conceive once she stop using them? She will not conceive. It is better if she first gives birth to a child and then starts using contraceptive methods….” (IDI04, 43 years woman, currently non-user of modern contraceptives, urban).*

Another reason was the urge to prove fertility:

*“If I had continued with studies and completed my secondary school education before using any contraceptive methods, I could have got a child first and then use the [contraceptive] methods. (* ***I:*** *why?) …. how could I have known that I was fertile while I was using pills or injectables?.......” (IDI02, 20 years old woman, currently non-user of modern contraceptives, rural).*

For some participants, the ability to access family planning to delay a first birth among students was not necessarily a good thing as it carried the potential of giving the student inappropriate confidence to engage in sexual activities.

*“…...when she [parent] sees that her child [=daughter] is going to start form one [=secondary school education] she takes her to the dispensary for implants. But I think it [=implants] makes the child to be confident and start engaging into a bad peer groups because she is confident that she already has implants…….” (FGD, 19 years-old woman, rural).*

### The type of contraceptive

The district reproductive and child health coordinator and district medical officer who oversee reproductive and child health services in the district reported that: ***“*** *It depends on the client’s choice” (IDI05, DRCHco).* However the DRCHco went on to say that in their experience the long-acting reversible methods (intrauterine devices and implants) were ideal for women who would like to delay their first birth and that they generally counselled for the use of these method types.

*For example a girl who is still studying cannot use injectables because she will get tired to visit for injections. So you advice her to use IUD or implant that can last for three to five years. Other clients receive advice from their peers and they come for injectables, but we counsel them that injectables are provided after every three months and you will get tired, you better use IUD” (IDI05, DRCHco).*

By contrast, some family planning providers in facilities said that they did not always recommend or provide long-acting reversible methods for delayers of first birth. The first reason was a request for early removal of the methods especially from newly married women. Some providers said they would not recommend long acting reversible methods to delayers of first birth who are currently unmarried to avoid the women to come to the facility asking to remove them before the removal date as soon as they get married.

*“… For delayers it is condoms, pills, and injectables……There are reasons for this, for example, you cannot provide implants to a woman who wants to delay a first pregnancy for one year because implants protect for three years.…….. Another client can tell you that when I inserted the implant I was not yet married. Now I am married and my husband needs a child…” (IDI01, FP provider, district hospital).*

*“.....When you ask most of them they say “once I get married I will stop using”, so you can advise her to use implants and find her coming back after a week for removal (=before removal date)” (IDI04, FP service provider, public health centre).*

A second reason was concern about a rapid return to fecundity. Some of the providers recommend pills as the most appropriate contraceptive method for delayers of first birth because of the perception that a return to fecundity would be quicker than other methods, including injectables.

*“I usually recommend them to use pills because she can conceive as soon as she stops taking them. The temporary side effects from injectables do delay a woman to conceive for up to six or nine months” (IDI04, FP service provider, public health centre).*

The majority of participants who took part in FGDs and women who took part in the IDIs agreed with this perspective:

*“....those who have not given birth should use pills. She can conceive immediately at any time once she stops...that is what we have said that anyone who has never been pregnant even once uses the pills, when she stops she will get a child (FGD, 39 years-old woman, urban).*

The third factor influencing the providers’ choice of method was logistical. The providers reported that many facilities experienced stock-outs and frequently had a limited method mix to offer clients.

*“Because injectables and pills are the most available methods, our clients choose injectables and pills” (IDI03, FP service provider, dispensary).*

An additional logistical constraint was that some providers have not received training in long-acting reversible methods and the methods were not ordered for their facilities. Clients were reported to be referred to other facilities with trained staff or were asked to wait for district staff who come to provide the service once or twice a year accompanied by staff from Marie-Stopes Tanzania.

*“…some clients say “I need implants”. I normally tell them that I have not received any training on how to insert implants and implants are not available here [=at this facility]. Therefore, I refer them to other facilities where they are provided” (IDI02, FP service provider, mission health centre).*

### Non-alignment among national policies for adolescents

Multiple policies and laws are in place to protect the rights of young people in Tanzania, including having access to family planning services from the age of 10 years and with confidentiality without the need for consent from parents, guardian or spousal except for people with severe mental disabilities and clients seeking permanent methods [26, 27]. The Tanzania child law of 2009 defines a child as anyone who is below the age of 18 years and according to the Tanzania education and training policy of 2014 [40] a girl who started primary school at the age of seven years, is likely to turn 18 while she is still at secondary school. Men who engage into sexual activities with a primary or secondary school student and impregnate or marry the school girl can face up to 30 years in prison [41]. Conversely, the minimum marriage age for girls in Tanzania according to the Tanzania marriage law of 1971 is 15 years with parental consent [28]. At the district level the actors implementing these laws are hindered by lack of clarity and consistency about age-restrictions. In the case of family planning this has direct implications on the issue of access, confidentiality and consent for young people.

#### Access, confidentiality and consent

Young women who visit facilities face minimum age limit and limited method choice. Family planning service providers at the district hospital were asked by the district hospital social welfare officer not to provide the service to any student or woman below 18 years before referring her to a district hospital social welfare for counselling. This pointed to a lack of confidentiality between clients and service providers, confusion about eligible age to receive contraception and the need for consent.

*“.....I normally tell them that when a child comes to you to seek [family planning services] while she has not yet conceived, if she is ten or eleven or twelve years, I tell them to first refer them to [me] social welfare officer so that I can understand what is her problem such that she wants to use the service and I advice her. Therefore, that is how we work closely with RCH department.....” (IDI07, DHSWO).*

*“….they are children according to the child law of 2009 that I use to protect them. So, once she is received [=by family planning providers] and she is under 18 years, even if she has four children to me she is a child and she is my client…” (IDI07, DHSWO).*

*“If I counsel a student and manage to make her to not get involved into contraceptive use while she is under 18 years and still not become pregnant, to me that is a big achievement.……If I wake up in the morning and find more than 20 children queuing for family planning services, in terms of behavioural change, I am a failure” (IDI07, DHSWO).*

In addition, family planning providers reported that for the clients brought by their parents or relatives, the parent or the relative explained the purpose of the visit and/or chose the contraception method. This pointed to a limited autonomy in decision-making to access the service and choice of specific contraception method among young women.

*“The mother is the one who explains “she is here for injectables”. What I then do is just explain to them about injectables [use] and so on. I do not ask her [the client brought by her mother] if she is willing to use or not…., when they arrive I just take it as they have agreed each other since home. …, from 14 years, 15 years up to 19 years, students” (IDI02, FP service provider, mission health centre).*

*“Those who are brought by their parents I only ask the parent that “this child is still young, has not decided yet and you have decided for her, do you really want your child to be given injectables? She replies “yes”. This means that she discussed with her at home and then brought her here. I provide her [the method] because she is brought by her mother. I cannot tell her that I won’t give you while she is the one who brought her here. She is confident that her child is grown up and is able to get involved into sexual activities. She came here so that she can avoid pregnancy”…… (IDI03, FP service provider, dispensary, rural).*

Lack of autonomy in the decision-making to use contraception for some young women and students was corroborated with FGDs and IDIs participants when sharing their experience on how the decision to access and use specific contraception for the first time was made.

*“……..I do not know what they discussed, I was just injected with the injectables……My sister told me that “let me take you to the dispensary because you are still a student. Perhaps you meet men and sleep with them, they will impregnate you, I do not want that, I want you to complete your studies”. Then I was taken to the dispensary and she talked to the facility staff and I was injected….when I say that I do not know anything it is because they went and talked alone, after they have talked and agreed, I was just injected with the injectables”.(IDI02, 20 years-old woman, currently non-user of modern contraceptives, rural).*

## Discussion

Our results suggest that being a student, being young, unmarried, or being in an unstable marriage were strongly perceived to be acceptable reasons for women using modern contraceptives to delay their first birth. However, long-acting reversible methods such as implants and intrauterine devices were perceived as inappropriate methods for delaying first birth. In addition, there was lack of clarity and consistency on acceptable age at first use of contraception and that had direct implications for access, autonomy in decision-making, confidentiality and consent for young people. A few participants disapproved use of any modern contraceptives for delaying first birth mainly due to: 1) the defined fertility concept- that there is an amount of limited fertility a woman has and if contraceptive methods were used, a woman might end up infertile; 2) proving woman’s fertility; and 3) lack of clan as extended families dissolve and nuclear style families become more prevalent you have to rely on your children rather than your clan.

Similar to a recent research in Kenya [42], several participants approved use of modern contraceptives despite acknowledging that religious beliefs prohibit their use. A more important emerging issue, and also reported in other settings [8, 15–17] was the varying views on age limit at first use of the modern methods, expressed by people at community and at facility level, despite participant’s commonly agreed views on decreasing age at first sex in their community. In southern Tanzania, the majority of delayers of first birth use injectables, pills and condoms [35] -methods reported to have high discontinuation rates [29, 43]. Consistent with previous studies [44, 45], fear of delayed return to fecundity, discontinuation of long-acting reversible contraceptive methods such as implants and intrauterine devices in order to become pregnant once a woman’s marital status changes were the main reasons for not recommending the methods to women who would like to delay their first birth. But this lack of method mix for delayers may also be related to the insufficient number of trained staff in long-acting reversible contraceptive methods in the study area –a common problem in low and middle income countries [17, 46, 47]. The recent Tanzania service provision assessment survey also found that long-acting reversible contraceptive methods were not widely available throughout the country and only 18% of facilities that offered any modern methods had at least one staff trained in family planning in 24 months before the survey [32].

Contrary to the Tanzanian national family planning policy, guideline and standards [26, 27] officials at district management and facility levels had contradictory interpretation of the policy with regards to young women, students and unmarried women. This was similar to challenges reported in previous studies in sub-Saharan Africa including Tanzania where consent requirement were imposed or women were expected to have given birth to at least one child before adoption of modern contraceptives [8, 16, 17, 19, 20]. In addition, in some families that supported delayers of first birth who were young or students to access contraception, the family members played a key role in decision making to access and choosing the method-an indication of lack of autonomy in decision-making among the young women. This may not be unique to Tanzania: a meta-ethnography on what influences adolescents’ contraceptives decision making in United States also found that parents made contraceptive decisions for some adolescents [48]. And it has also been reported from Uganda that family planning providers also chose contraceptive methods for some young people - again pointing to lack of autonomy in decision making among young women [21]. Conversely, not all women lacked autonomy in decision making to use modern contraceptives since it was reported that women married in an unstable marriage were using the methods to delay their first birth while learning their husband’s behaviours such as intimate physical violence, or for financial and economical stability reasons. Findings from a recent study conducted in Ghana also found that women who justified wife beating in one or more instances were less likely to use modern contraceptives suggesting that women who did not justify wife beating’s behaviour were more likely to use modern contraceptives [49]. About 70 % of nulliparous women over 33 years in the UK were reported to be using modern contraceptives for delaying their first birth due to reasons concerning their relationships with husbands or partners [50] also consistent with our finding that using modern contraception was acceptable for women in unstable relationships.

The views of the few participants who disapproved the use of modern contraception to delay first birth were similar to a recent study in Uganda, where contraception was perceived to be for spacing subsequent pregnancies, and access to the contraception was affected by the women’s overwhelming fear of permanent infertility [51]. In addition, their views were similar to the findings from a study conducted in India where women who were pressured to prove their fertility as soon as they got married were less likely to use modern contraceptives [22]. This may not be unique to India: a previous study [52] had reported that the value of children in African countries also places high expectations on young women to start childbearing in order to maintain family lineage and for the children to provide labour. However, in sub-Saharan Africa including Tanzania, as school enrolment and income-earning opportunities for women increase [53], prevalence of sexual activities among adolescents aged 10–19 years [8] and government increased commitment to provide family planning services [24, 25], there is likely to be an increasing demand for the use of modern contraceptive for delaying first birth. For example, a recent study in Southern Tanzania found that all of the women who wanted to delay their first pregnancy had demand for modern contraceptives, yet only 59 % of the women were currently using modern contraceptives [35]. Since the majority of the women who would like to delay their first birth are young adolescents and unmarried women [35] who are less likely to access a range of these methods, service provision to this group needs clarity and consistency on age limits and appropriate contraception method.

This qualitative study has some limitations. While all health facility levels were represented the number of interviewees from health facilities was relatively small; nonetheless, across all responder groups saturation was reached for the questions posed. Every effort was taken to minimise the effect of methodological issues that can introduce bias in qualitative studies, for example maintaining reflexivity throughout data collection to reduce the influence of researchers on responses, providing clear selection criteria for interviewees, and analysis being carried out by a fluent Swahili and English speaker to preserve the original meaning of responses to the extent possible. Despite these efforts respondent bias cannot be discounted, particularly among health staff directly involved in provision of family planning services.

## Conclusions

In conclusion, our study demonstrates that in southern Tanzania it is acceptable to use modern contraceptives to delay first birth if a woman is a primary or secondary school student, young, unmarried, or in an unstable marriage. These women face challenges related to restrictions by age and method imposed by stakeholders. There is a need for a well-communicated policy on minimum age and appropriate contraception methods for the delayers of first birth.

## Abbreviations

DHS: Demographic health surveys
DHSWO: District Hospital based Social Welfare Officer
DMO: District Medical Officer
DRCHco: District Reproductive and Child Health Co-ordinator
FGDs: Focus group discussions
FP: Family planning
IDIs: In-depth Interviews
IUCDs: Intrauterine (copper contained) devices and systems
LARCs: Long-acting reversible contraceptives
RCH: Reproductive and child health
